# (4*R*,5*S*)-5-Benzyl-4-isopropyl-1,3,4-oxadiazinan-2-one

**DOI:** 10.1107/S1600536808013056

**Published:** 2008-05-10

**Authors:** Lacey D. Addison, Delvis D. Dore, Shawn R. Hitchcock, Gregory M. Ferrence

**Affiliations:** a CB 4160, Department of Chemistry, Illinois State University, Normal, IL 61790, USA

## Abstract

The title compound, C_13_H_18_N_2_O_2_, is an N_4_-isopropyl-l-phenyl­alanine-based oxadiazinanone. Although the two mol­ecules in the asymmetric unit are oriented appropriately for hydrogen bonding, the distance between the donor and acceptor atoms is large enough to support only weak, if any, hydrogen bonding. The absolute configuration is known based on the known starting compounds in the synthetic procedure.

## Related literature

For related literature, see: Burgeson *et al.* (2004 ▶); Casper, Blackburn *et al.* (2002 ▶); Casper, Burgeson *et al.* (2002 ▶); Casper & Hitchcock (2003 ▶); Dore *et al.* (2006 ▶); Ferrence *et al.* (2003 ▶); Hitchcock *et al.* (2004 ▶); Hitchcock *et al.* (2001 ▶); Squire *et al.* (2005 ▶); Szczepura *et al.* (2004 ▶); Bruno *et al.* (2004 ▶).
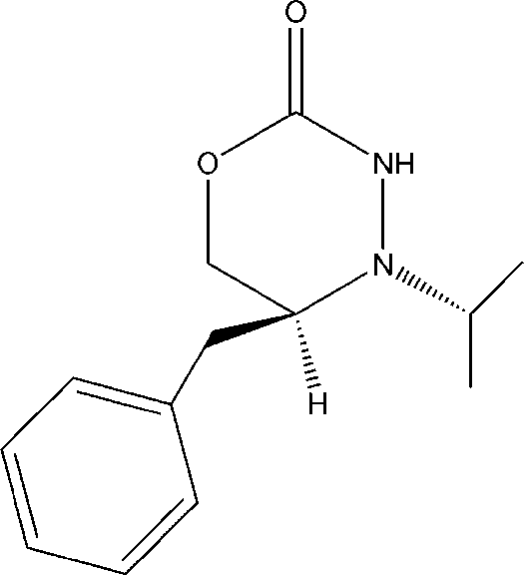


## Experimental

### 

#### Crystal data

C_13_H_18_N_2_O_2_
*M* *_r_* = 234.29
Orthorhombic, 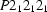

*a* = 9.6423 (14) Å
*b* = 11.4974 (17) Å
*c* = 22.600 (3) Å
*V* = 2505.5 (6) Å^3^
*Z* = 8
Mo *K*α radiation
μ = 0.09 mm^−1^
*T* = 100 (2) K
0.43 × 0.23 × 0.23 mm

#### Data collection

Bruker SMART APEX CCD diffractometer
Absorption correction: multi-scan *SADABS* in *SAINT-Plus* (Bruker, 2003 ▶) *T*_min_ = 0.965, *T*_max_ = 0.981
25602 measured reflections
3499 independent reflections
3403 reflections with *I* > 2σ(*I*)
*R*_int_ = 0.041

#### Refinement

*R*[*F* ^2^ > 2σ(*F* ^2^)] = 0.060
*wR*(*F* ^2^) = 0.133
*S* = 1.32
3499 reflections
307 parameters
H-atom parameters constrained
Δρ_max_ = 0.37 e Å^−3^
Δρ_min_ = −0.25 e Å^−3^

### 

Data collection: *SMART* (Bruker, 2003 ▶); cell refinement: *SAINT-Plus* (Bruker, 2003 ▶); data reduction: *SAINT-Plus*; program(s) used to solve structure: *SIR2004* (Burla *et al.*, 2005 ▶); program(s) used to refine structure: *SHELXL97* (Sheldrick, 2008 ▶); molecular graphics: *ORTEP-3 for Windows* (Farrugia, 1997 ▶) and *Mercury* (Macrae *et al.*, 2006 ▶); software used to prepare material for publication: *WinGX* (Farrugia, 1999 ▶) and *publCIF* (Westrip, 2008 ▶).

## Supplementary Material

Crystal structure: contains datablocks global, I. DOI: 10.1107/S1600536808013056/sg2243sup1.cif

Structure factors: contains datablocks I. DOI: 10.1107/S1600536808013056/sg2243Isup2.hkl

Additional supplementary materials:  crystallographic information; 3D view; checkCIF report

**Fig. 5.**
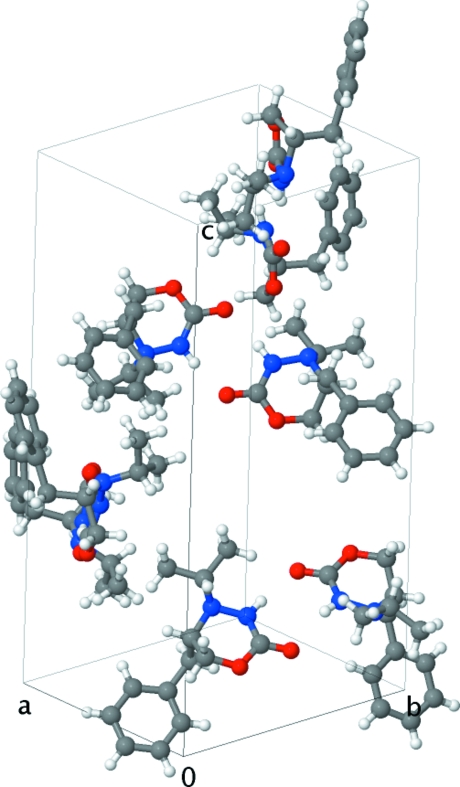
Jmol enhanced figure of I. The default view shows the basic unit-cell contents. The pair of molecules forming the asymmetric unit and the putative H-bonding pair of molecules may be highlighted when viewing the active enhanced figure.

Enhanced figure: interactive version of Fig. 5

## Figures and Tables

**Fig. 1.**
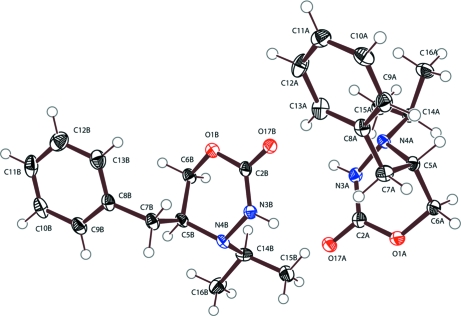
The molecular structure of compound (I), with the atomic numbering scheme. Displacement ellipsoids are drawn at the 50% probability level.

**Fig. 2.**
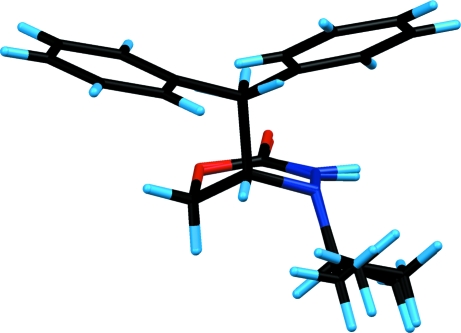
A Mercury view of the asymmetric unit of (I) highlighting the 2.83 Å N3A—O17B and 2.89 Å N3B—O17A donor to acceptor separations which support only weak, if any, hydrogen bonding.

**Fig. 3.**
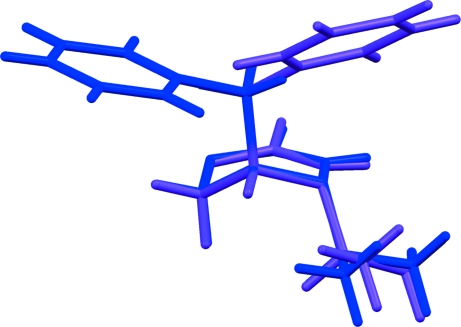
A Mercury overlay of the two independent molecules in the asymmetric unit of (I) with H atoms shown in light blue.

**Fig. 4.**
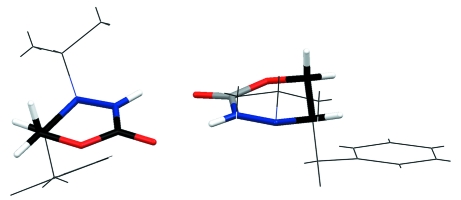
A Mercury overlay of the two independent molecules in the asymmetric unit of (I) with one molecule shown in blue and one shown in violet.

## supplementary crystallographic information

### Comment

The synthesis (Hitchcock *et al.*, 2001), conformational analysis (Casper, Blackburn *et al.*, 2002; Burgeson *et al.* 2004), and asymmetric applications (Casper & Hitchcock, 2003; Casper, Burgeson *et al.*, 2002; Ferrence *et al.* 2003; Hitchcock *et al.* 2004; Hitchcock *et al.* 2001; Squire *et al.* 2005; Szczepura *et al.* 2004) of 3,4,5,6-tetrahydro-2*H*-1,3,4-oxadiazinan-2-ones have only thoroughly been studied in the last ten years. We have been interested in synthesizing new oxadiazinanones for use as chiral auxillaries in aldol addition reactions. We synthesized the title compound in order to study the conformation that the heterocycle adopts. Herein we report the single-crystal X-ray structure analysis of the N_4_-isopropyl-*L*-phenylalanine based oxiadiazinanone.

Other oxadiazinanones have been reported and studied, but the title compound is one of few studied that is not substituted at the N3 position. Other oxadiazinanone structures (Burgeson *et al.*, 2004; Casper, Blackburn *et al.*, 2002; Casper, Burgeson *et al.*, 2002; Ferrence *et al.*, 2003; Hitchcock *et al.*, 2001, 2004) are substituted with a carbonyl at the N3 position. These N3 substituted oxadiazinanones adopt a twist-boat conformation, as does the title compound. This is also consistent with related oxadiazinanones not substituted at the N3 position (Szczepura *et al.*, 2004). The C7B—C5B—N4B—C14B torsion angle is 159.1 (2)°, and the C7A—C5A—N4A—C14A torsion angle is 155.5 (2)°. Previously reported oxadiazinanones with no substitution at the N3 position have torsion angles between 161.79–163.16°. A *Mogul* (Bruno *et al.* 2004) geometry check showed all non-H bond angles and distances to be normal. The molecular structure (Fig. 1.) of I includes two independent molecules in the asymmetric unit. The oxadiazinanone moieties are essentially isostructural. The primary difference between the two molecules is the orientation of the benzyl group attached to C5A/B (Figs. 2. and 3.). The respective -56.9 (3)° N4A—C5A—C7B—C8A and -175.5 (2)° N4A—C5A—C7B—C8A torsion angles quantify this difference.

Hydrogen-bonding interactions usually appear to play a key role in the crystal packing of oxadiazinanones (Szczepura *et al.*, 2004). However, it may be that the optimal crystal packing simply happens to yield an arrangement of molecules which are suggestive of a hydrogen bonding motif. That is packing forces other than formation of the weak H-bonding fortuitously lead to the motif. In the title compound, the 2.83 Å N3A—O17B and 2.89 Å N3B—O17A donor to acceptor separations are large enough to support only weak, if any, hydrogen bonding (Fig 4.). This interaction is further illustrated in the Jmol enhanced figure (Fig. 5).

### Experimental

The title compound was prepared as previously reported (Dore *et al.* (2006)).

### Refinement

All non-H atoms were refined anisotropically without disorder. All H atoms were initially identified through difference Fourier syntheses then removed and included in the refinement in the riding-model approximation (C–H = 0.95, 0.98, 0.99 and 1.00 Å for Ar–H, CH_3_ and CH_2_ and CH; N–H = 0.88 Å; *U*_iso_(H) = 1.2Ueq(C) except for methyl groups, where *U*_iso_(H) = 1.5Ueq(C)). In the absence of significant anomalous scattering effects, Friedel pairs were merged.

### Crystal data

**Table:**

C_13_H_18_N_2_O_2_	*F*_000_ = 1008
*M**_r_* = 234.29	*D*_x_ = 1.242 Mg m^−^^3^
Orthorhombic, *P*2_1_2_1_2_1_	Mo *K*α radiation λ = 0.71073 Å
Hall symbol: P 2ac 2ab	Cell parameters from 8817 reflections
*a* = 9.6423 (14) Å	θ = 2.3–30.5º
*b* = 11.4974 (17) Å	µ = 0.09 mm^−^^1^
*c* = 22.600 (3) Å	*T* = 100 (2) K
*V* = 2505.5 (6) Å^3^	Block, colourless
*Z* = 8	0.43 × 0.23 × 0.23 mm

### Data collection

**Table:**

Bruker SMART APEX CCD diffractometer	3403 reflections with *I* > 2σ(*I*)
Radiation source: sealed tube	*R*_int_ = 0.041
Monochromator: graphite	θ_max_ = 28.3º
ω scans	θ_min_ = 1.8º
Absorption correction: multi-scanSADABS in SAINT-Plus (Bruker, 2003)	*h* = −12→12
*T*_min_ = 0.965, *T*_max_ = 0.981	*k* = −15→15
25602 measured reflections	*l* = −29→30
3499 independent reflections	

### Refinement

**Table:**

Refinement on *F*^2^	H-atom parameters constrained
Least-squares matrix: full	*w* = 1/[σ^2^(*F*_o_^2^) + (0.0413*P*)^2^ + 1.6549*P*] where *P* = (*F*_o_^2^ + 2*F*_c_^2^)/3
*R*[*F*^2^ > 2σ(*F*^2^)] = 0.060	(Δ/σ)_max_ < 0.001
*wR*(*F*^2^) = 0.133	Δρ_max_ = 0.37 e Å^−^^3^
*S* = 1.32	Δρ_min_ = −0.25 e Å^−^^3^
3499 reflections	Extinction correction: none
307 parameters	

### Special details

**Table:**

Geometry. All e.s.d.'s (except the e.s.d. in the dihedral angle between two l.s. planes) are estimated using the full covariance matrix. The cell e.s.d.'s are taken into account individually in the estimation of e.s.d.'s in distances, angles and torsion angles; correlations between e.s.d.'s in cell parameters are only used when they are defined by crystal symmetry. An approximate (isotropic) treatment of cell e.s.d.'s is used for estimating e.s.d.'s involving l.s. planes.

### Fractional atomic coordinates and isotropic or equivalent isotropic displacement parameters (Å^2^)

**Table:**

	*x*	*y*	*z*	*U*_iso_*/*U*_eq_	
O1A	0.5275 (2)	0.74524 (18)	0.71574 (9)	0.0200 (4)	
C2A	0.5986 (3)	0.7897 (3)	0.76134 (13)	0.0176 (6)	
O17A	0.6692 (2)	0.87684 (18)	0.75304 (9)	0.0213 (4)	
N3A	0.5897 (2)	0.7389 (2)	0.81457 (10)	0.0170 (5)	
H3A	0.6512	0.7603	0.8412	0.02*	
N4A	0.4904 (2)	0.6534 (2)	0.83257 (10)	0.0154 (5)	
C5A	0.3806 (3)	0.6483 (2)	0.78779 (12)	0.0165 (5)	
H5A	0.3265	0.5752	0.7943	0.02*	
C6A	0.4435 (3)	0.6429 (3)	0.72638 (13)	0.0191 (6)	
H6A1	0.5015	0.5723	0.7227	0.023*	
H6A2	0.3687	0.6385	0.6964	0.023*	
C7A	0.2806 (3)	0.7517 (3)	0.79456 (13)	0.0206 (6)	
H7A1	0.3317	0.8247	0.7865	0.025*	
H7A2	0.2059	0.7447	0.7647	0.025*	
C8A	0.2163 (3)	0.7588 (3)	0.85530 (13)	0.0189 (6)	
C9A	0.0827 (3)	0.7173 (3)	0.86541 (14)	0.0232 (6)	
H9A	0.031	0.686	0.8333	0.028*	
C10A	0.0233 (3)	0.7206 (3)	0.92131 (15)	0.0263 (7)	
H10A	−0.0681	0.6921	0.9273	0.032*	
C11A	0.0976 (3)	0.7656 (3)	0.96826 (14)	0.0251 (7)	
H11A	0.0584	0.7669	1.0068	0.03*	
C12A	0.2293 (3)	0.8086 (3)	0.95870 (15)	0.0281 (7)	
H12A	0.2802	0.8407	0.9908	0.034*	
C13A	0.2878 (3)	0.8053 (3)	0.90287 (15)	0.0249 (7)	
H13A	0.3786	0.8354	0.8971	0.03*	
C14A	0.5541 (3)	0.5392 (2)	0.84671 (13)	0.0180 (6)	
H14A	0.5723	0.4953	0.8093	0.022*	
C15A	0.6894 (3)	0.5566 (3)	0.88013 (14)	0.0250 (7)	
H15A	0.7547	0.5999	0.8552	0.037*	
H15B	0.7291	0.4808	0.8902	0.037*	
H15C	0.6714	0.6006	0.9165	0.037*	
C16A	0.4514 (4)	0.4714 (3)	0.88512 (15)	0.0285 (7)	
H16A	0.3644	0.4606	0.8633	0.043*	
H16B	0.4331	0.5149	0.9216	0.043*	
H16C	0.4908	0.3952	0.895	0.043*	
O1B	0.8672 (2)	0.98233 (18)	0.94720 (9)	0.0218 (5)	
C2B	0.7980 (3)	0.9425 (2)	0.89999 (13)	0.0175 (5)	
O17B	0.7047 (2)	0.87193 (19)	0.90751 (9)	0.0221 (5)	
N3B	0.8282 (3)	0.9856 (2)	0.84613 (11)	0.0187 (5)	
H3B	0.7824	0.955	0.8163	0.022*	
N4B	0.9257 (3)	1.0754 (2)	0.83144 (11)	0.0177 (5)	
C5B	0.9584 (3)	1.1386 (2)	0.88641 (13)	0.0188 (6)	
H5B	1.0451	1.1841	0.8792	0.023*	
C6B	0.9879 (3)	1.0537 (2)	0.93632 (14)	0.0200 (6)	
H6B1	1.0676	1.0036	0.9256	0.024*	
H6B2	1.0123	1.0971	0.9727	0.024*	
C7B	0.8430 (3)	1.2258 (3)	0.90152 (14)	0.0216 (6)	
H7B1	0.8272	1.2779	0.8673	0.026*	
H7B2	0.7558	1.1832	0.9094	0.026*	
C8B	0.8812 (3)	1.2979 (2)	0.95530 (14)	0.0205 (6)	
C9B	0.9766 (4)	1.3882 (3)	0.95059 (15)	0.0261 (7)	
H9B	1.0129	1.4084	0.9129	0.031*	
C10B	1.0197 (4)	1.4496 (3)	1.00054 (16)	0.0291 (7)	
H10B	1.0845	1.5114	0.9968	0.035*	
C11B	0.9680 (4)	1.4202 (3)	1.05520 (17)	0.0325 (8)	
H11B	0.9978	1.4613	1.0894	0.039*	
C12B	0.8727 (4)	1.3309 (3)	1.06053 (16)	0.0341 (8)	
H12B	0.8368	1.3108	1.0983	0.041*	
C13B	0.8296 (4)	1.2707 (3)	1.01068 (15)	0.0286 (7)	
H13B	0.7636	1.2099	1.0146	0.034*	
C14B	1.0493 (3)	1.0225 (3)	0.80199 (13)	0.0207 (6)	
H14B	1.0973	0.9692	0.8303	0.025*	
C15B	1.0028 (4)	0.9540 (3)	0.74778 (15)	0.0255 (6)	
H15D	0.9394	0.8919	0.76	0.038*	
H15E	1.084	0.9198	0.7283	0.038*	
H15F	0.9553	1.0062	0.7201	0.038*	
C16B	1.1483 (4)	1.1180 (3)	0.78295 (15)	0.0320 (8)	
H16D	1.1788	1.1619	0.8178	0.048*	
H16E	1.101	1.1705	0.7554	0.048*	
H16F	1.229	1.0833	0.7634	0.048*	

### Atomic displacement parameters (Å^2^)

**Table:**

	*U*^11^	*U*^22^	*U*^33^	*U*^12^	*U*^13^	*U*^23^
O1A	0.0219 (10)	0.0205 (10)	0.0176 (9)	−0.0022 (9)	−0.0014 (8)	0.0017 (8)
C2A	0.0151 (12)	0.0162 (13)	0.0215 (14)	0.0042 (11)	−0.0003 (11)	−0.0015 (11)
O17A	0.0237 (10)	0.0186 (10)	0.0217 (10)	−0.0034 (9)	−0.0026 (9)	0.0017 (9)
N3A	0.0147 (11)	0.0186 (11)	0.0178 (11)	−0.0011 (10)	−0.0048 (9)	−0.0007 (9)
N4A	0.0146 (11)	0.0122 (10)	0.0193 (11)	0.0005 (9)	−0.0011 (9)	0.0018 (9)
C5A	0.0141 (12)	0.0171 (12)	0.0182 (13)	−0.0037 (11)	−0.0020 (10)	0.0000 (11)
C6A	0.0199 (14)	0.0189 (13)	0.0185 (14)	−0.0034 (12)	−0.0037 (11)	−0.0001 (11)
C7A	0.0178 (13)	0.0234 (15)	0.0207 (14)	0.0031 (12)	−0.0033 (11)	0.0025 (12)
C8A	0.0175 (13)	0.0149 (13)	0.0243 (14)	0.0059 (11)	−0.0035 (11)	0.0008 (11)
C9A	0.0196 (14)	0.0234 (14)	0.0267 (15)	−0.0036 (13)	−0.0054 (12)	−0.0021 (13)
C10A	0.0217 (15)	0.0255 (15)	0.0317 (16)	−0.0036 (13)	0.0020 (13)	0.0049 (13)
C11A	0.0286 (16)	0.0246 (15)	0.0221 (14)	0.0080 (14)	0.0000 (13)	0.0029 (12)
C12A	0.0248 (15)	0.0331 (18)	0.0265 (16)	0.0082 (14)	−0.0089 (13)	−0.0110 (14)
C13A	0.0147 (13)	0.0271 (16)	0.0329 (17)	0.0028 (12)	−0.0013 (12)	−0.0063 (13)
C14A	0.0232 (14)	0.0139 (12)	0.0169 (13)	0.0039 (11)	−0.0021 (11)	0.0013 (10)
C15A	0.0276 (16)	0.0234 (15)	0.0239 (15)	0.0076 (13)	−0.0055 (13)	0.0013 (12)
C16A	0.0364 (18)	0.0229 (15)	0.0262 (15)	−0.0040 (14)	−0.0032 (14)	0.0086 (13)
O1B	0.0249 (11)	0.0187 (10)	0.0218 (10)	−0.0014 (9)	−0.0009 (9)	0.0005 (9)
C2B	0.0183 (13)	0.0134 (12)	0.0207 (13)	0.0045 (11)	0.0003 (11)	−0.0028 (11)
O17B	0.0254 (11)	0.0210 (10)	0.0197 (10)	−0.0059 (9)	0.0037 (9)	−0.0019 (8)
N3B	0.0194 (12)	0.0183 (12)	0.0185 (11)	−0.0044 (10)	−0.0018 (10)	0.0001 (9)
N4B	0.0174 (11)	0.0136 (10)	0.0222 (12)	−0.0026 (9)	−0.0005 (9)	0.0021 (9)
C5B	0.0183 (13)	0.0146 (12)	0.0233 (14)	−0.0044 (11)	−0.0033 (11)	0.0017 (11)
C6B	0.0190 (13)	0.0160 (12)	0.0249 (15)	0.0004 (11)	−0.0045 (12)	0.0010 (11)
C7B	0.0202 (13)	0.0159 (13)	0.0286 (15)	0.0020 (11)	−0.0040 (12)	−0.0003 (12)
C8B	0.0190 (13)	0.0141 (13)	0.0284 (15)	0.0063 (11)	−0.0065 (12)	−0.0013 (11)
C9B	0.0283 (16)	0.0179 (14)	0.0319 (17)	0.0011 (13)	−0.0076 (14)	0.0023 (13)
C10B	0.0257 (16)	0.0160 (14)	0.046 (2)	0.0012 (13)	−0.0127 (15)	−0.0024 (14)
C11B	0.0362 (19)	0.0232 (15)	0.0382 (19)	0.0108 (15)	−0.0117 (16)	−0.0114 (14)
C12B	0.0343 (18)	0.0376 (19)	0.0305 (17)	0.0087 (16)	0.0041 (15)	−0.0045 (15)
C13B	0.0257 (15)	0.0237 (17)	0.0365 (18)	0.0026 (14)	0.0035 (14)	−0.0045 (14)
C14B	0.0161 (13)	0.0246 (14)	0.0215 (14)	0.0050 (12)	0.0007 (11)	0.0062 (12)
C15B	0.0250 (14)	0.0223 (14)	0.0291 (15)	−0.0023 (13)	0.0086 (12)	−0.0022 (13)
C16B	0.0276 (16)	0.045 (2)	0.0236 (16)	−0.0147 (16)	0.0054 (13)	−0.0039 (15)

### Geometric parameters (Å, °)

**Table:**

O1A—C2A	1.339 (3)	O1B—C2B	1.339 (3)
O1A—C6A	1.449 (3)	O1B—C6B	1.444 (4)
C2A—O17A	1.226 (4)	C2B—O17B	1.223 (4)
C2A—N3A	1.340 (4)	C2B—N3B	1.346 (4)
N3A—N4A	1.431 (3)	N3B—N4B	1.435 (3)
N3A—H3A	0.88	N3B—H3B	0.88
N4A—C5A	1.466 (3)	N4B—C5B	1.474 (4)
N4A—C14A	1.484 (3)	N4B—C14B	1.494 (4)
C5A—C6A	1.516 (4)	C5B—C6B	1.519 (4)
C5A—C7A	1.538 (4)	C5B—C7B	1.536 (4)
C5A—H5A	1	C5B—H5B	1
C6A—H6A1	0.99	C6B—H6B1	0.99
C6A—H6A2	0.99	C6B—H6B2	0.99
C7A—C8A	1.508 (4)	C7B—C8B	1.517 (4)
C7A—H7A1	0.99	C7B—H7B1	0.99
C7A—H7A2	0.99	C7B—H7B2	0.99
C8A—C13A	1.385 (4)	C8B—C13B	1.383 (5)
C8A—C9A	1.393 (4)	C8B—C9B	1.391 (4)
C9A—C10A	1.388 (4)	C9B—C10B	1.394 (5)
C9A—H9A	0.95	C9B—H9B	0.95
C10A—C11A	1.381 (5)	C10B—C11B	1.374 (5)
C10A—H10A	0.95	C10B—H10B	0.95
C11A—C12A	1.380 (5)	C11B—C12B	1.383 (5)
C11A—H11A	0.95	C11B—H11B	0.95
C12A—C13A	1.383 (5)	C12B—C13B	1.386 (5)
C12A—H12A	0.95	C12B—H12B	0.95
C13A—H13A	0.95	C13B—H13B	0.95
C14A—C15A	1.521 (4)	C14B—C16B	1.517 (4)
C14A—C16A	1.530 (4)	C14B—C15B	1.524 (5)
C14A—H14A	1	C14B—H14B	1
C15A—H15A	0.98	C15B—H15D	0.98
C15A—H15B	0.98	C15B—H15E	0.98
C15A—H15C	0.98	C15B—H15F	0.98
C16A—H16A	0.98	C16B—H16D	0.98
C16A—H16B	0.98	C16B—H16E	0.98
C16A—H16C	0.98	C16B—H16F	0.98
			
C2A—O1A—C6A	117.9 (2)	C2B—O1B—C6B	117.4 (2)
O17A—C2A—O1A	118.6 (3)	O17B—C2B—O1B	118.9 (3)
O17A—C2A—N3A	121.9 (3)	O17B—C2B—N3B	121.9 (3)
O1A—C2A—N3A	119.5 (3)	O1B—C2B—N3B	119.1 (3)
C2A—N3A—N4A	126.7 (2)	C2B—N3B—N4B	128.0 (2)
C2A—N3A—H3A	116.7	C2B—N3B—H3B	116
N4A—N3A—H3A	116.7	N4B—N3B—H3B	116
N3A—N4A—C5A	108.3 (2)	N3B—N4B—C5B	107.4 (2)
N3A—N4A—C14A	113.1 (2)	N3B—N4B—C14B	109.5 (2)
C5A—N4A—C14A	114.3 (2)	C5B—N4B—C14B	114.0 (2)
N4A—C5A—C6A	110.2 (2)	N4B—C5B—C6B	110.4 (2)
N4A—C5A—C7A	110.7 (2)	N4B—C5B—C7B	110.8 (2)
C6A—C5A—C7A	111.9 (2)	C6B—C5B—C7B	113.0 (3)
N4A—C5A—H5A	108	N4B—C5B—H5B	107.5
C6A—C5A—H5A	108	C6B—C5B—H5B	107.5
C7A—C5A—H5A	108	C7B—C5B—H5B	107.5
O1A—C6A—C5A	110.1 (2)	O1B—C6B—C5B	109.9 (2)
O1A—C6A—H6A1	109.6	O1B—C6B—H6B1	109.7
C5A—C6A—H6A1	109.6	C5B—C6B—H6B1	109.7
O1A—C6A—H6A2	109.6	O1B—C6B—H6B2	109.7
C5A—C6A—H6A2	109.6	C5B—C6B—H6B2	109.7
H6A1—C6A—H6A2	108.2	H6B1—C6B—H6B2	108.2
C8A—C7A—C5A	112.9 (2)	C8B—C7B—C5B	111.1 (2)
C8A—C7A—H7A1	109	C8B—C7B—H7B1	109.4
C5A—C7A—H7A1	109	C5B—C7B—H7B1	109.4
C8A—C7A—H7A2	109	C8B—C7B—H7B2	109.4
C5A—C7A—H7A2	109	C5B—C7B—H7B2	109.4
H7A1—C7A—H7A2	107.8	H7B1—C7B—H7B2	108
C13A—C8A—C9A	117.8 (3)	C13B—C8B—C9B	118.4 (3)
C13A—C8A—C7A	121.5 (3)	C13B—C8B—C7B	120.9 (3)
C9A—C8A—C7A	120.7 (3)	C9B—C8B—C7B	120.5 (3)
C10A—C9A—C8A	121.4 (3)	C8B—C9B—C10B	120.8 (3)
C10A—C9A—H9A	119.3	C8B—C9B—H9B	119.6
C8A—C9A—H9A	119.3	C10B—C9B—H9B	119.6
C11A—C10A—C9A	119.7 (3)	C11B—C10B—C9B	119.7 (3)
C11A—C10A—H10A	120.1	C11B—C10B—H10B	120.2
C9A—C10A—H10A	120.1	C9B—C10B—H10B	120.2
C12A—C11A—C10A	119.4 (3)	C10B—C11B—C12B	120.1 (3)
C12A—C11A—H11A	120.3	C10B—C11B—H11B	119.9
C10A—C11A—H11A	120.3	C12B—C11B—H11B	119.9
C11A—C12A—C13A	120.6 (3)	C11B—C12B—C13B	119.9 (3)
C11A—C12A—H12A	119.7	C11B—C12B—H12B	120
C13A—C12A—H12A	119.7	C13B—C12B—H12B	120
C12A—C13A—C8A	121.0 (3)	C8B—C13B—C12B	121.0 (3)
C12A—C13A—H13A	119.5	C8B—C13B—H13B	119.5
C8A—C13A—H13A	119.5	C12B—C13B—H13B	119.5
N4A—C14A—C15A	110.2 (2)	N4B—C14B—C16B	109.5 (3)
N4A—C14A—C16A	107.8 (2)	N4B—C14B—C15B	109.5 (2)
C15A—C14A—C16A	109.9 (2)	C16B—C14B—C15B	109.3 (3)
N4A—C14A—H14A	109.7	N4B—C14B—H14B	109.5
C15A—C14A—H14A	109.7	C16B—C14B—H14B	109.5
C16A—C14A—H14A	109.7	C15B—C14B—H14B	109.5
C14A—C15A—H15A	109.5	C14B—C15B—H15D	109.5
C14A—C15A—H15B	109.5	C14B—C15B—H15E	109.5
H15A—C15A—H15B	109.5	H15D—C15B—H15E	109.5
C14A—C15A—H15C	109.5	C14B—C15B—H15F	109.5
H15A—C15A—H15C	109.5	H15D—C15B—H15F	109.5
H15B—C15A—H15C	109.5	H15E—C15B—H15F	109.5
C14A—C16A—H16A	109.5	C14B—C16B—H16D	109.5
C14A—C16A—H16B	109.5	C14B—C16B—H16E	109.5
H16A—C16A—H16B	109.5	H16D—C16B—H16E	109.5
C14A—C16A—H16C	109.5	C14B—C16B—H16F	109.5
H16A—C16A—H16C	109.5	H16D—C16B—H16F	109.5
H16B—C16A—H16C	109.5	H16E—C16B—H16F	109.5
			
C6A—O1A—C2A—O17A	−179.4 (2)	C6B—O1B—C2B—O17B	172.3 (2)
C6A—O1A—C2A—N3A	−0.1 (4)	C6B—O1B—C2B—N3B	−11.2 (4)
O17A—C2A—N3A—N4A	166.0 (3)	O17B—C2B—N3B—N4B	174.2 (3)
O1A—C2A—N3A—N4A	−13.3 (4)	O1B—C2B—N3B—N4B	−2.2 (4)
C2A—N3A—N4A—C5A	−12.3 (4)	C2B—N3B—N4B—C5B	−17.0 (4)
C2A—N3A—N4A—C14A	115.5 (3)	C2B—N3B—N4B—C14B	107.2 (3)
N3A—N4A—C5A—C6A	47.0 (3)	N3B—N4B—C5B—C6B	46.5 (3)
C14A—N4A—C5A—C6A	−80.2 (3)	C14B—N4B—C5B—C6B	−75.0 (3)
N3A—N4A—C5A—C7A	−77.3 (3)	N3B—N4B—C5B—C7B	−79.5 (3)
C14A—N4A—C5A—C7A	155.5 (2)	C14B—N4B—C5B—C7B	159.1 (2)
C2A—O1A—C6A—C5A	35.5 (3)	C2B—O1B—C6B—C5B	41.9 (3)
N4A—C5A—C6A—O1A	−60.1 (3)	N4B—C5B—C6B—O1B	−61.0 (3)
C7A—C5A—C6A—O1A	63.5 (3)	C7B—C5B—C6B—O1B	63.7 (3)
N4A—C5A—C7A—C8A	−56.9 (3)	N4B—C5B—C7B—C8B	−175.5 (2)
C6A—C5A—C7A—C8A	179.9 (2)	C6B—C5B—C7B—C8B	60.0 (3)
C5A—C7A—C8A—C13A	79.7 (4)	C5B—C7B—C8B—C13B	−99.3 (3)
C5A—C7A—C8A—C9A	−99.6 (3)	C5B—C7B—C8B—C9B	76.3 (3)
C13A—C8A—C9A—C10A	−0.9 (5)	C13B—C8B—C9B—C10B	0.3 (5)
C7A—C8A—C9A—C10A	178.4 (3)	C7B—C8B—C9B—C10B	−175.4 (3)
C8A—C9A—C10A—C11A	−0.2 (5)	C8B—C9B—C10B—C11B	0.4 (5)
C9A—C10A—C11A—C12A	1.1 (5)	C9B—C10B—C11B—C12B	−0.6 (5)
C10A—C11A—C12A—C13A	−1.0 (5)	C10B—C11B—C12B—C13B	0.2 (5)
C11A—C12A—C13A—C8A	−0.2 (5)	C9B—C8B—C13B—C12B	−0.7 (5)
C9A—C8A—C13A—C12A	1.1 (5)	C7B—C8B—C13B—C12B	175.0 (3)
C7A—C8A—C13A—C12A	−178.3 (3)	C11B—C12B—C13B—C8B	0.5 (5)
N3A—N4A—C14A—C15A	40.3 (3)	N3B—N4B—C14B—C16B	176.4 (2)
C5A—N4A—C14A—C15A	164.9 (2)	C5B—N4B—C14B—C16B	−63.2 (3)
N3A—N4A—C14A—C16A	160.2 (2)	N3B—N4B—C14B—C15B	56.5 (3)
C5A—N4A—C14A—C16A	−75.2 (3)	C5B—N4B—C14B—C15B	176.9 (2)
